# Animated, video entertainment-education to improve vaccine confidence globally during the COVID-19 pandemic: an online randomized controlled experiment with 24,000 participants

**DOI:** 10.1186/s13063-022-06067-5

**Published:** 2022-02-19

**Authors:** Simiao Chen, Sebastian Forster, Juntao Yang, Fengyun Yu, Lirui Jiao, Jennifer Gates, Zhuoran Wang, Haitao Liu, Qiushi Chen, Pascal Geldsetzer, Peixin Wu, Chen Wang, Shannon McMahon, Till Bärnighausen, Maya Adam

**Affiliations:** 1 Heidelberg Institute of Global Health (HIGH), Faculty of Medicine and University Hospital, Heidelberg University, Heidelberg, Germany; 2 Chinese Academy of Medical Sciences and Peking Union Medical College, Beijing, China; 3 State Key Laboratory of Medical Molecular Biology, Institute of Basic Medical Sciences, Chinese Academy of Medical Sciences & Peking Union Medical College, Beijing, China; 4 Department of Industrial Engineering, Tsinghua University, Beijing, China; 5 Reed College, Portland, OR USA; 6 Icahn School of Medicine at Mount Sinai, New York, NY USA; 7 The Harold and Inge Marcus Department of Industrial and Manufacturing Engineering, The Pennsylvania State University, University Park, PA USA; 8 Department of Medicine, Stanford University School of Medicine, Stanford, CA USA; 9 Peking Union Medical College Hospital, Beijing, China; 10 National Clinical Research Center for Respiratory Diseases, Beijing, China; 11 Department of Pulmonary and Critical Care Medicine, Center of Respiratory Medicine, China–Japan Friendship Hospital, Beijing, China; 12 Department of Global Health and Population, Harvard T.H. Chan School of Public Health, Boston, MA USA; 13 Africa Health Research Institute (AHRI), Somkhele, KwaZulu-Natal South Africa; 14 Department of Pediatrics, Stanford University School of Medicine, Stanford, CA USA

**Keywords:** COVID-19, Rrandomized controlled trial, Vaccine hesitancy, Vaccine acceptance, Protocol, Hope, List experiment, Vaccine confidence

## Abstract

**Background:**

Science-driven storytelling and entertainment-education (E-E) media demonstrate potential for promoting improved attitudes and behavioral intent towards health-related practices. Months after the outbreak of coronavirus disease 2019 (COVID-19), emerging research highlights the essential role of interventions to improve public confidence in the COVID-19 vaccine. To improve vaccine confidence, we designed three short, animated videos employing three research-informed pedagogical strategies. These can be distributed globally through social media platforms, because of their wordless and culturally accessible design. However, the effectiveness of short, animated storytelling videos, deploying various pedagogic strategies, needs to be explored across different global regions.

**Methods/design:**

The present study is a multi-site, parallel group, randomized controlled trial (RCT) comparing the effectiveness of (i) a storytelling-instructional-humor approach, (ii) a storytelling-analogy approach, (iii) a storytelling-emotion-focused approach, and (iv) no video. For our primary outcomes, we will measure vaccine hesitancy, and for secondary outcomes, we will measure behavioral intent to seek vaccination and hope. Using online platforms, we will recruit 12,000 participants (aged 18–59 years) from the USA and China, respectively, yielding a total sample size of 24,000.

**Discussion:**

This trial uses innovative online technology, reliable randomization algorithms, validated survey instruments, and list experiments to establish the effectiveness of three short, animated videos employing various research-informed pedagogical strategies. Results will be used to scientifically support the broader distribution of these short, animated video as well as informing the design of future videos for rapid, global public health communication.

**Trial registration:**

German Clinical Trials Register DRKS #00023650. Date of registration: 2021/02/09.

**Supplementary Information:**

The online version contains supplementary material available at 10.1186/s13063-022-06067-5.

## Administrative information

**Table:** 

Title	Animated, video entertainment-education to improve vaccine confidence globally during the COVID-19 pandemic: an online randomized controlled experiment with 24,000 participants
**Trial registration**	DRKS #00023650
**Funding**	This study is funded by an Alexander von Humboldt Foundation (Prize recipient: Dr. Till Bärnighausen) and the Sino-German Center for Research Promotion (Project C-0048), which is funded by the German Research Foundation (DFG) and the National Natural Science Foundation of China (NSFC).
**Name and contact information for the trial sponsor**	https://www.humboldt-foundation.de/en/https://www.dfg.de/en/dfg_profile/head_office/dfg_abroad/beijing/index.html
**Role of sponsor**	The funders will have no role in the collection, management, analysis, and interpretation of data; writing of the report or submission decisions.

## Background and rationale

In the midst of the COVID-19 pandemic, research highlights the critical role of interventions to increase vaccine confidence, even before vaccines for COVID-19 become universally available [1, 2]. The WHO has dubbed vaccine hesitancy one of the greatest threats to global health [3], yet we are lacking interventions that effectively promote vaccine confidence in ways that are accessible and globally scalable. Many existing interventions have focused on providing information in the form of scientific data, using a didactic approach. This strategy has proven ineffective for key target audiences [4].

Designing effective interventions for a broad range of target audiences has become even more important during the COVID-19 pandemic as misinformation has spread rapidly around the world. Negative claims about vaccines often appeal to the emotions of the target audiences, eliciting vaccine doubt and hesitancy. This observation—that emotion-focused messages resonate and affect health behaviors [4, 5]—can also potentially be used to the advantage of health communicators. Research suggests that activating positive emotions, like hope and altruism, can actually bolster vaccine education interventions [5].

Other promising pedagogical strategies, including the use of instructional humor [6], analogies [7, 8], and storytelling, could also be leveraged to promote vaccine confidence, and researchers have advocated for a transdisciplinary approach to successful health communication on vaccines [4]. By integrating different fields of expertise, including those outside of academia—like entertainment and marketing—we may be able to design more effective vaccine promotion interventions. Animated E-E videos, developed using transdisciplinary approaches, could be an especially effective method for distributing evidence-based health messages globally through social media platforms [9, 10].

To improve vaccine confidence, we designed three videos, employing three research-informed pedagogical strategies: (i) a storytelling-instructional-humor approach (ii) a storytelling-analogy approach (iii) a storytelling-emotion-focused approach.

The first prototype video was released on Stanford Medicine’s YouTube channel (https://youtu.be/Ut_6GInouYg) on October 19th, 2020, and was viewed 27,290 times within the first 3 weeks. This video belongs to a collection of science-driven, storytelling COVID-19 animations that have already reached several million viewers globally. Because all of the videos contain no spoken words, these interventions can be rapidly distributed to global audiences without translation. Video interventions could play an important role in broadly disseminating health information during the COVID-19 pandemic, but there is a need to evaluate the efficacy of such interventions for improving vaccine confidence.

A secondary aim of the intervention videos is to convey hope, a measurable parameter that research suggests is related to improved health, psychosocial, and academic outcomes [11]. Recent research even suggests that leveraging positive emotions, including hope, may be leveraged as part of COVID-19 vaccine education interventions [5]. Hope has been defined as the perceived capacity to build pathways towards our goals and motivate ourselves to use those pathways. Hope theory proposed by Snyder [11] has been likened to the theories of optimism, self-efficacy, and self-esteem. Especially during the COVID-19 pandemic, a period characterized by widespread emotional distress [12], bolstering hope could have meaningful positive effects on the mental health of the global public.

The aim of this study is to systematically evaluate the efficacy of animated, short video interventions, with different pedagogical approaches, for (a) reducing vaccine hesitancy, (b) increasing behavioral intent to get vaccinated, and (c) increasing hope. Here, we propose an online experiment in which the video intervention and a survey will be randomly ordered and assigned to 24,000 participants between the age of 18 and 59, living in China or the USA. These two countries represent a considerable population in the world, which are culturally different from the other and have also managed the pandemic in significantly different ways [13]. Results will be used to scientifically support the ongoing distribution of these interventions as well as optimizing the design of future animated, E-E videos for public health communication.

## Objective

Our study aims to achieve the following objectives. To:

1. Establish the effectiveness of each of the intervention videos in reducing COVID-19 vaccine hesitancy (primary objective).
2. Establish the effectiveness of each of the intervention videos in increasing behavioral intent towards COVID-19 vaccination.
3. Establish the effectiveness of each of the intervention videos in increasing participants’ level of hope.

## Methods: participants, interventions, and outcomes

### Study setting

This trial will be conducted online, using the SPIRIT reporting guidelines [14]. For the United States, we will use the research platform created and managed by Prolific Academic Ltd (ProA: https://www.prolific.co/) to recruit participants and an online web platform Gorilla (www.gorilla.sc) to host and deploy our study; for China, we will use Kurundata, which recruits members in a variety of ways, including through its own platform (https://www.kurundata.com/), partnerships with other websites, and encouraging registered members to recruit new members through the popular mobile application WeChat (Appendix S1).

### Trial design

**Fig. 1:**
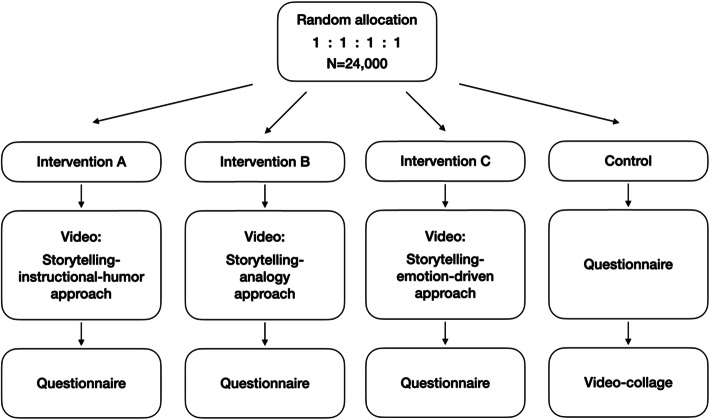
Study design with 24,000 participants randomized to receive the video and questionnaire (treatment arm) or questionnaire and video (control arm)

Our study is a multi-site, parallel group, randomized controlled trial (RCT) comparing the effectiveness of each of the short intervention videos with each other and with no video (the control condition). Via the online research platforms, participants will be randomly assigned to four intervention arms (Video A: storytelling-instructional-humor approach, Video B: storytelling-analogy approach, Video C: storytelling-emotion-driven approach) and a control arm (no video). Participants will be randomized at a 1:1:1:1 ratio (Fig. 1). In each trial arm, there is a questionnaire survey, arranged in the following order. *Intervention arm a*: participants will receive the storytelling-instructional-humor video, followed by the survey. *Intervention arm b*: participants will receive the storytelling-analogy video, followed by the survey. *Intervention arm c*: participants will receive the storytelling-emotion-focused video, followed by the survey. *Control arm*: participants will first receive the survey. After survey responses are submitted, participants in the control arm will be given access to the video interventions arranged in a single loop (to ensure post-trial access to treatment).

### Eligibility criteria

To be eligible, participants must be between the age of 18 and 59, living in China or the USA. Since this video is wordless and culturally inclusive, therefore accessible to participants of all language and cultural groups. The English questionnaire will be translated into Chinese, so participants from the USA must have reading competency in English, and participants from China must have reading competency in Chinese to complete the trial questionnaire. Those who do not sign the consent form or drop out will be excluded from the data analysis.

### Ethical approval

Ethics approval was obtained from the Stanford University IRB on January 12th, 2021, protocol #59503, and from the Chinese Academy of Medical Sciences & Peking Union Medical College IRB on March 10th, 2021.

### Who will obtain informed consent?

Participants must preview an information and consent form before they can begin the survey. The form explains the purpose of the study, the risks and benefits of the research, and how to contact the study investigators (or the Stanford University ethics review board). By clicking the link, participants consent to participate in our study and will be redirected to the Gorilla or Kurundata platform, where additional information is given. Participants can exercise their freedom to participate (or decline participation) at recruitment or at any point during the study.

### Criteria for discontinuing or modifying allocated interventions

Since this is a minimal risk study of an online educational video intervention, we do not anticipate needing to discontinue or modify the allocated interventions during the course of the study. Participants can withdraw from the study at any time and participants will not be compensated for incomplete surveys. Those participants who choose not to sign the consent form or fail to complete the experiment in its entirety will be excluded from the data analysis.

## Interventions

### Intervention description

The video interventions are a short (2–3-min) animated E-E videos about vaccines. All videos use a storytelling approach, but each video differs slightly in its pedagogical approach. *Intervention arm a* will view a video that uses a storytelling-instructional-humor approach. *Intervention arm b* will view a video that uses a storytelling-analogy approach and *intervention arm c* will view a video that uses a storytelling-emotion-focused approach. The videos were developed with input from advisors at the Immunization Action Coalition, Vaccinate Your Family (formerly, Every Child by Two), the Stanford University Pediatrics Dept. Division of Infectious Diseases, the Icahn School of Medicine, the University of Texas Rio Grande Valley School of Medicine, and the Heidelberg Institute of Global Health. The video interventions have no words, speech, or text, but incorporate soundtracks consisting of music and sound effects. The videos demonstrate how COVID-19 has impacted lives around the world and how a vaccine could catalyze a partial return to pre-pandemic lifestyles. The videos are designed for universal reach and optimized for release on social media. The interventions can be viewed at the links below in countries that allow access to YouTube:

Video A: https://youtu.be/ap8xpyREaTc

Video B: https://youtu.be/fYYBJ0d6gl0

Video C: https://youtu.be/WH5KUhGtfa8

### Explanation for the choice of comparators

The comparators are similar-length E-E videos, all animated in the same styles by the same animator. They all convey the same message (i.e., vaccines work) using a storytelling approach, without the use of spoken or written language. Each intervention arm video uses a slightly different pedagogical approach. The control arm receives no video intervention. Comparing the *intervention arms a, b* and *c* with the control arm will allow us to quantify the effect of each intervention on the primary and secondary outcomes. Comparing *intervention arms a, b* and *c* with each other will allow us to explore the differential effect of these pedagogical approaches on the primary and secondary outcomes. We will use list experiments, also referred to as the unmatched count technique [15], to eliminate social desirability bias regarding vaccine-seeking behavioral intent. For the list experiments, we will use the control list as the comparator.

### Outcomes

#### Primary outcome measures

Our primary outcome is vaccine hesitancy (including COVID-19 vaccine hesitancy). We will ask participants how much they agree or disagree with statements related to perceived vaccine safety as well as their attitudes, preferences, beliefs, and hesitancies regarding regular vaccines and the COVID-19 vaccine, respectively [16]. In the statistical analysis process, we will use nine questions to measure the degree of vaccine hesitancy in general and seven questions to measure the COVID-19 vaccine hesitancy specifically. Then we will normalize the final score to a range of 1–5 for both measures for comparison, which higher scores indicate higher degrees of vaccine hesitancy. These questions are shown in Table 1 and the data elicited from this survey will enable us to achieve objective 1.

**Table 1:** The (COVID-19) vaccine hesitancy items

Vaccine hesitancy	COVID-19 vaccine hesitancy
L1. Vaccines are important for my health	L1. COVID-19 vaccines are important for my health
L2. Vaccines are effective	L2. COVID-19 vaccines are effective
L3. Having myself vaccinated is important for the health of others in my community	L3. Having myself vaccinated with a COVID­19 vaccine is important for the health of others in my community
L4. All vaccines offered by the government programme in my community are beneficial.	L4. All COVID-19 vaccines offered by the government program in my community are beneficial
L5. New vaccines carry more risks than older vaccines	L5. COVID-19 vaccines from other countries carry more risks than vaccines from my country
L6. I trust the information I receive about shots	L6. The information I receive about COVID-19 vaccines from the vaccine program is reliable and trustworthy
L7. Getting vaccines is a good way to protect myself from disease	L7. Getting COVID-19 vaccines is a good way to protect myself from COVID-19
L8. I am able to openly discuss my concerns about shots with my doctor	L8. Generally, I do what my doctor or health care provider recommends about COVID-19 vaccines for myself
L9. I am concerned about serious adverse effects of vaccines	L9. I am concerned about serious adverse effects of COVID-19 vaccines
L10. People do not need vaccines for diseases that are not common anymore	L10. I do not need COVID-19 vaccines if it’s not a pandemic anymore
L11. I believe that many of the illnesses shots prevent are severe	L11. I am concerned that COVID-19 vaccines might not prevent the disease
L12. It is better to get fewer vaccines at the same time	L12. I am concerned that COVID-19 vaccines might not be safe
L13. People get more shots than are good for them	
L14. It is better to develop immunity by getting sick than to get a shot	

*Note: the survey tool was designed based on [16], which requires the level of agreement from “strongly disagree” to “strongly agree”

#### Secondary outcome measures

Our secondary outcomes include participants’ behavioral intent to get vaccinated as well as participants’ level of hope. In order to reduce the social desirability bias often associated with direct questioning about sensitive items [15], we will use the unmatched count or list randomization approach to devise a series of list experiments [17]. Four list experiments will be used, as shown in Table 2. For each experiment, the control group will receive a list of three items. Participants are asked how many items they are likely to do in the coming months without stating which ones they chose. The treatment group will get the identical item lists but with one additional “sensitive” item relating to their behavioral intent to get vaccinated or encourage their loved ones to do so. For example, imagining that the control group select 1 out of the 3 items on average while the treatment group select 1.3 out of the 4 items, with the assumption that the average acceptance of these two cohorts is the same, we can conclude that the prevalence of participants who would get vaccinated against COVID-19, is 20%. We have designed the list of experiments in accordance with published best practices [17], and these data will be used to assess objective 2.

**Table 2:** List experiments in the trial

**List 1: COVID vaccine—self uptake**	
1. Brush my teeth at least twice daily	
2. Begin learning a new language	
3. Smoke cigarettes or vape	
4. Get vaccinated against COVID-19 when the vaccine is available for me^*^	
**List 2: COVID vaccine—recommendation**	
1. Recommend a show or movie to my friend	
2. Encourage a friend to seek routine dental care	
3. Allow a friend to drive home even though I think they may have had too much to drink	
4. Encourage a friend or family member to get vaccinated against COVID-19 when the vaccine is available for them^*^	
**List 3: routine vaccine—self uptake**	
1. Wash my hands before eating	
2. Take up a new sport	
3. Have unprotected sex with someone who is not my long-term partner	
4. Get a routine vaccine (for example, flu vaccine, tetanus booster shots, hepatitis B vaccine) if the doctor recommends it^*^	
**List 4: routine vaccine—recommendation**	
1. Try to get my family to eat more fruits and vegetables	
2. Perform a routine check of the batteries in our smoke detectors	
3. Encourage a friend to get a tattoo or body piercing	
4. Encourage a friend or family member to get a routine vaccine (for example, flu vaccine, tetanus booster shots, hepatitis B vaccine) if the doctor recommends it^*^	

Note: In each trial arm, both groups will receive four lists. For each list, the control group will get the first three items only; the treatment group will receive the three items and the fourth sensitive item, indicated by an asterisk (*). Each list experiment will be preceded by the question: “In the coming 3 months, how many of the following things are you likely to do? I do not need to know which of these things you are likely to do, just how many.”

Second, we will assess participants’ level of hope using the Adult Hope Scale [18], a 12-item scale measuring participants’ level of hope (Table 3). The Adult Hope Scale is comprised of two subscales relating to Snyder’s cognitive model of hope: (1) Agency (goal-directed energy) and (2) Pathways (planning to accomplish goals) [11]. The 12 items include 4 Agency items, 4 Pathways items, and 4 distractors. Participants are asked to respond using an 8-point Likert-type scale ranging from Definitely False to Definitely True. As recommended in the literature, we will refer to the scale as “The Future Scale” within the survey experiment.

**Table 3:** The future scale

Items	Response
I1. I can think of many ways to get out of a jam.	1. = Definitely false
I2. I energetically pursue my goals.	2. = Mostly false
I3. I feel tired most of the time.	3. = Somewhat false
I4. There are lots of ways around any problem.	4. = Slightly false
I5. I am easily downed in an argument.	5. = Slightly true
I6. I can think of many ways to get the things in life that are important to me.	6. = Somewhat true
I7. I worry about my health.	7. = Mostly true
I8. Even when others get discouraged, I know I can find a way to solve the problem.	8. = Definitely true
I9. My past experiences have prepared me well for my future.	
I10. I’ve been pretty successful in life.	
I11. I usually find myself worrying about something.	
I12. I meet the goals that I set for myself.	

Note: Items 2, 9, 10, and 12 make up the agency subscale. Items 1, 4, 6, and 8 make up the pathway subscale. Researchers can either examine results at the subscale level or combine the two subscales to create a total hope score

We also aim to measure the difference of the outcomes above between the three *intervention arms a* (storytelling-instructional-humor), *b* (storytelling-analogy), and *c* (storytelling emotion-focused). These data will be used to assess objective 3.

### Sample size

To calculate the sample size needed for pairwise comparison between three groups, we used a one-way analysis of variance (ANOVA). The formula is as follows.

$$ {n}_A=\left({\sigma}_A^2+\frac{\sigma_B^2}{\kappa}\right){\left(\frac{Z_{1-\frac{\alpha }{\tau }}+{Z}_{1-\beta }}{\mu_A-{\mu}_B}\right)}^2 $$

where *κ*, the matching ratio, is equal to 1 in our study; *μ*_*A*_ and *μ*_*B*_ are the means of group A and group B; *σ*_*A*_ and *σ*_*B*_ are the standard deviations; *α* = 0.05 and *β* = 0.2 are the type-I and type-II error, respectively; Z is the quantile function; and *τ* = 2 is the number of comparisons to be made. For vaccine hesitancy (and COVID-19 vaccine hesitancy), we assume that the means of two arms are 2.00 and 2.01, which represents the level of vaccine acceptance of this arm (1 means total acceptance and 5 means complete refusal), the standard deviations are the same, 0.10. To detect a difference of 0.01 between the vaccine hesitancy between arms, we can calculate the total minimal sample size is *n*_*A*_ = *n*_*B*_ = *n*_*C*_ = *n*_*D*_ = 1570, so the *N* = 6280. To test the sensitivity of the result, we can change our assumption and increase the standard deviation to 0.12, i.e., *σ*_*A*_ = *σ*_*B*_ = 0.12. Then we have *n*_*A*_ = *n*_*B*_ = *n*_*C*_ = *n*_*D*_ = 2260 and *N* = 9040. To achieve a higher level of accuracy, we will recruit 12,000 participants in each country for this study.

### Recruitment and implementation

We will recruit participants from the ProA and Kurundata platforms. Study participants will be compensated $1.30 for completing the survey at ProA and 5 RMB at Kurundata. ProA or Kurundata will make the reward payment to study participants. To complete the survey, a potential study participant must open an account on ProA or Kurundata and provide his or her personal information. Participants must agree to ProA’s or Kurundata’s data privacy terms and conditions. ProA or Kurundata will assign each participant a unique, anonymized ID. Because ProA and Kurundata handle the interaction between the study investigators and participants, the participants will be anonymous to the study investigators. ProA and Kurundata are both research platforms with access to large numbers of study participants, enabling us to achieve the desired sample size for this study.

### Assignment of interventions: allocation

All participants who give consent for participation and who fulfill the inclusion criteria will be randomized. After they complete the consent form, Gorilla and Kurundata will use a web-based randomization algorithm to randomly allocate participants to the intervention arm a, intervention arm b, intervention arm c, or control arm (sequence generation) at a 1:1:1:1 ratio. The randomization algorithm involves a web-based algorithm using random permutation, which is independent from the researchers who will recruit patients and implement the protocol. Once an arm of participants is fully recruited, subsequent participants will be randomly assigned to the remaining groups until all arms are fully recruited.

### Assignment of interventions: blinding

The study investigators and those involved in the data analyses and statistics will be blinded to the group allocation.

### Data collection and management

#### Plans for assessment and collection of outcomes

The study investigators will be responsible for data collection. Data will be collected on either the Gorilla or Kurundata platforms. The data retrieved from the platforms will be anonymous. Data downloaded will be stored on an encrypted and secure server. The data will be deleted two years after the study has been completed.

#### Plans to promote participant retention and completion

Participants will be automatically timed out from the online platform if they take more than 45 min to complete the study. Though participants can withdraw the study at any time, they will not be compensated for incomplete survey participation. Incomplete data will be excluded from our analyses. Since the participants are anonymous to us, there is no way to initiate follow-up in the time limit.

#### Data management

Data will be collected on the Gorilla or Kurundata platforms. Third parties except for ProA or Kurundata will not have access to the data. The data will be downloaded and safely stored for statistical analysis on a computing system maintained by the University of Heidelberg in Germany.

#### Confidentiality

Because of the anonymized participant IDs, the study investigators can never meet or know the identity of the study participants. The study investigators will only have access to the participants’ anonymized ID and no other personal or confidential information, and the data will be deleted 2 years after the study has been completed. The study investigators will keep this information confidential.

### Statistical methods

#### Descriptive measures

We will use descriptive statistics to obtain summaries of the demographic data (age, sex, education status, country of residence, etc.).

#### Primary outcomes

For each participant, we will calculate their (COVID-19) vaccine hesitancy score based on their survey responses. Let $\overline{K_k}$ denote the mean vaccine hesitancy for each trial arm, where *k* ∈ {*a*, *b*, *c*} such that *a* represents the intervention arm a (video with storytelling-instructional-humor approach), *b* represents the intervention arm b (video with storytelling-analogy approach), *c* represents the intervention arm c (video with storytelling-emotion-focused approach), and *d* represents the control arm. To evaluate the effectiveness of the video, we will use ANOVA for continuous endpoints and *χ*^2^ test for binary endpoints to assess the statistical significance of the difference between the control arm and the treatment arms. To investigate the factors that affect the endpoints, we will use generalized linear mixed models (GLMM) for the analysis of our endpoints. We use ordinary least squares (OLS) regression for our continuous endpoints; we will use (modified) Poisson regression for our binary endpoints; we will use negative binomial regression for our count endpoints. The reason for the choice of modified Poisson regression for our binary endpoints is that this analysis has good statistical properties and generates risk ratios, which are far easier and safer to interpret than the effect size measures generated by alternative methods (such as odds ratios or marginal effects) [19–22].

#### Secondary outcomes

For the vaccination plan in the list experiment, we will calculate the mean score for the control list and treatment list, denoted by $\overline{C_i}$ and $\overline{T_i}$ respectively, where *i* is the index of the list. Then we can calculate the mean difference between the control list and treatment list within each trial arm, which is considered as the participants’ behavioral intent to get vaccinated in this trial arm, denoted as $\overline{D_{ik}}=\overline{T_{ik}}-\overline{C_{ik}}$. Analogous to difference-in-difference analyses, we can identify the effect of each treatment.

For other secondary outcomes, we can use the same statistical procedure described above to assess the effectiveness of our E-E video. We will use R statistical software to undertake the analysis.

#### Interim analyses

No interim analyses are planned.

#### Methods for additional analyses (e.g., subgroup analyses)

Since we will conduct the trial in the USA and China, we will conduct both country-specific and cross-country pooled analyses for further comparison.

#### Methods in analysis to handle protocol non-adherence and any statistical methods to handle missing data

Participants who decide to withdraw from the trial will not be replaced

#### Plans to give access to the full protocol, participant level data, and statistical code

This document is the full protocol. Anyone interested in other data or documentation should contact the corresponding author.

### Oversight and monitoring

#### Composition of the coordinating center and trial steering committee

The trial will be overseen by a trial steering committee (TSC). The TSC will have an independent chairperson and members but also includes the trial collaborators. Two TSC meetings are planned.

#### Adverse event reporting and harms

As this is an online survey, there is very minimal risk for study-related injury or harm. We will present non-threatening video interventions and questions about vaccine attitudes. There are no foreseeable risks to participating in the online study.

#### Ancillary and post-trial care

No ancillary and post-trial care is planned.

#### Dissemination plans

The results of this study will be disseminated through presentations at international conferences and publications in peer-reviewed journals. Results will be used by the study collaborators and their institutions (Stanford School of Medicine, Heidelberg University, Chinese Academy of Medical Sciences, and Peking Union Medical College) to improve the design and universal appeal of future educational and health promotion videos. All investigators who meet authorship criteria will be included as co-authors and anyone who contributed, but does not meet the criteria for authorship, will be acknowledged. No professional author services will be used.

## Discussion

Global government and health authorities have actively engaged in efforts to combat the COVID-19 pandemic, including imposing a variety of strict lockdown policies on regions and countries. Also, since the World Health Organization (WHO) is currently orchestrating the global campaign to fight against the spread of the SARS-CoV-2 virus, the progress of vaccination has dramatically accelerated and it is foreseeable that numerous safe and effective vaccines will come to market by 2022 [23]. However, previous research has indicated that vaccine availability does not guarantee vaccine acceptance, especially given global variations in cultural and educational backgrounds [24]. In this study, we propose using animated, E-E videos to decrease general vaccine hesitancy globally and to evaluate their effectiveness using three innovative approaches.

First, we will use the latest online technology to conduct our multi-site, parallel, randomized controlled trial. ProA, the online platform, enables researchers to connect with individuals around the world who are interested in participating in online studies [25]. Kurundata fills a similar function in China. Second, we will host and deploy our study on the Gorilla platform, which is an experiment builder that provides users with the tools for undertaking online behavioral research. The Gorilla randomization algorithm, which demonstrates the platform’s capability to implement innovative trial designs, will guide us in randomly assigning participants at two levels: (1). Participants will be randomized to the storytelling-instructional-humor video arm, the storytelling-analogy video arm, the storytelling-emotion-focused video arm or no video arm (2). Within each arm, participants will be randomized to control list or treatment list within the list experiment portion of the survey.

Third, we will deploy list experiments to reduce the social desirability bias associated with sensitive questions such as the intention to seek vaccination. Prior research highlights the documented discrepancies between publicly declared vaccine intentions and privately held reservations, including safety and efficacy concerns [24]. Therefore, we have designed the list experiment to minimize social desirability bias toward the behavioral intention questions.

This study is expected to establish the effectiveness of short, animated, E-E videos, using different pedagogical approaches, for improving vaccine confidence. We hope to document a reduction in vaccine hesitancy and guide future E-E video development strategies to support education and health communication campaigns globally.

## Trial status

This protocol was submitted on March 15th, 2021, and resubmitted on Jan 5, 2022. Recruitment for the trial and all data collection will have been completed by February 2022.

## Supplementary Information

**Additional file 1:.** Appendix S1

## Data Availability

Data will be collected and stored on the Gorilla and Kurundata platform. The study investigators own and have complete control of the research data, which can be accessed at any time. For statistical analysis, the data will be downloaded and safely stored on a computing system maintained by the University of Heidelberg.

## References

[1] De Figueiredo A, Simas C, Karafillakis E, Paterson P, Larson HJ (2020). Mapping global trends in vaccine confidence and investigating barriers to vaccine uptake: a large-scale retrospective temporal modelling study. Lancet..

[2] Salmon DA, Dudley MZ (2020). It is time to get serious about vaccine confidence. Lancet..

[3] World Health Organization (2020). Ten threats to global health in 2019.

[4] Laura J, Tarunjose K, Angelina K, Mojisola O, Enisa S, Valentine V, Fleur V, Lise B, Corinne V (2018). How Storytelling Can Combat Vaccine Hesitancy: a Transdisciplinary Approach. Transdiscipl Insights..

[5] Chou WYS, Budenz A (2020). Considering Emotion in COVID-19 Vaccine Communication: Addressing Vaccine Hesitancy and Fostering Vaccine Confidence. Health Commun..

[6] Wanzer MB, Frymier AB, Irwin J (2010). An explanation of the relationship between instructor humor and student learning: Instructional humor processing theory. Commun Educ..

[7] Gazzinelli MF, Lobato L, Matoso L, Avila R, Marques RD, Brown AS (2010). Health Education through Analogies: Preparation of a Community for Clinical Trials of a Vaccine against Hookworm in an Endemic Area of Brazil. Plos Neglect Trop D.

[8] Heywood D (2002). The Place of Analogies in Science Education. Camb J Educ..

[9] Adam M, Bärnighausen T, SA MM (2020). Design for extreme scalability: A wordless, globally scalable COVID-19 prevention animation for rapid public health communication. J Global Health.

[10] Vandormael A, Adam M, Greuel M, Bärnighausen T. An entertainment-education approach to prevent COVID-19 spread: study protocol for a multi-site randomized controlled trial. Trials. 2020;21(1)1025. 10.1186/s13063-020-04942-7 PMC7737415 33323130

[11] Snyder CR (2002). Hope theory: Rainbows in the mind. Psychol Inq..

[12] Pfefferbaum B, North CS (2020). Mental Health and the Covid-19 Pandemic. N Engl J Med..

[13] Wang C, Tripp C, Sears SF, Xu L, Tan Y, Zhou D, Ma W, Xu Z, Chan NA, Ho C, Ho R (2021). The impact of the COVID-19 pandemic on physical and mental health in the two largest economies in the world: a comparison between the United States and China. J Behav Med..

[14] Chan AW, Tetzlaff JM, Gotzsche PC, Altman DG, Mann H, Berlin JA, et al. SPIRIT 2013 explanation and elaboration: guidance for protocols of clinical trials. Bmj-Brit Med J. 2013;346(jan08 15). 10.1136/bmj.e7586. 10.1136/bmj.e7586 PMC3541470 23303884

[15] Krumpal I (2013). Determinants of social desirability bias in sensitive surveys: a literature review. Qual Quant..

[16] Larson HJ, Jarrett C, Schulz WS, Chaudhuri M, Zhou Y, Dube E, Schuster M, MacDonald N, Wilson R, SAGE Working Group on Vaccine Hesitancy (2015). Measuring vaccine hesitancy: The development of a survey tool. Vaccine..

[17] Glynn AN (2013). What Can We Learn with Statistical Truth Serum?. Public Opin Q..

[18] Snyder CR, Harris C, Anderson JR, Holleran SA, Irving LM, Sigmon ST, Yoshinobu L, Gibb J, Langelle C, Harney P (1991). The will and the ways: development and validation of an individual-differences measure of hope. J Pers Soc Psychol..

[19] Zou G (2004). A modified poisson regression approach to prospective studies with binary data. Am J Epidemiol..

[20] Zou G, Donner A (2013). Extension of the modified Poisson regression model to prospective studies with correlated binary data. Stat Methods Med Res..

[21] Ortblad K, Kibuuka Musoke D, Ngabirano T, Nakitende A, Magoola J, Kayiira P, Taasi G, Barresi LG, Haberer JE, McConnell MA, Oldenburg CE, Bärnighausen T (2017). Direct provision versus facility collection of HIV self-tests among female sex workers in Uganda: a cluster-randomized controlled health systems trial. PLoS Med..

[22] Yapa HM, De Neve J-W, Chetty T, Herbst C, Post FA, Jiamsakul A (2020). The impact of continuous quality improvement on coverage of antenatal HIV care tests in rural South Africa: Results of a stepped-wedge cluster-randomised controlled implementation trial. PLoS Med..

[23] Callaway E (2020). The race for coronavirus vaccines: a graphical guide. Nature..

[24] Dror AA, Eisenbach N, Taiber S, Morozov NG, Mizrachi M, Zigron A, Srouji S, Sela E (2020). Vaccine hesitancy: the next challenge in the fight against COVID-19. Eur J Epidemiol..

[25] Prolific Academic Research Platform. https://www.prolific.co/. Accessed 2 Sept 2022.

