# Supplementary material for: Animated, video entertainment-education to improve vaccine confidence globally during the COVID-19 pandemic: an online randomized controlled experiment with 24,000 participants
Source: Trials. 2022 Feb 19;23:161. doi: 10.1186/s13063-022-06067-5 (PMC8857749; doi:10.1186/s13063-022-06067-5)
Supplement: Supplementary file 1 — Additional file 1:. Appendix S1 [file 13063_2022_6067_MOESM1_ESM.docx]

**DESCRIPTION:** You are invited to participate in a research study on the outbreak of COVID-19. The purpose of the research is to get a better understanding of what people know about COVID-19. If you agree to take part in this study, you will be asked to watch a 2.30 min video about the COVID-19 outbreak and complete survey questions. Or you will be asked to first complete the survey questions and then watch a video about the COVID-19 outbreak. The chance of you receiving either the video or survey questions first is 50%. We will ask questions about your age, sex, country of residence, educational status, your knowledge of COVID-19, and your media usage.

During the study, data generated from your participation in the study will be collected by the ProA platform and de-identified. We refer to all such data as “Your Study Data,” which will be specifically regulated in the EU/EEA under the General Data Protection Regulation (the “GDPR”). Your de-identified Study Data may be processed or used for the following purposes, which we refer to, collectively, as “Data Processing”: to carry out the study, to confirm the accuracy of the study, to monitor that the study complies with applicable laws as well as best practices developed by the research community. The following entities and organizations may engage in Data Processing of Your Study Data:

- the study team, including other people who, and organizations that, assist the study team:
- the study sponsor: Yvonne Maldonado, MD
- the ethics committee or institutional review board that approved this study; and
- domestic and foreign regulatory agencies and government officials who have a duty to monitor or oversee studies like this one.
- We have entered into a data transfer agreement with Stanford University which is based on standard contractual clauses approved by the European Commission and ensures an adequate protection for Your Study Data. You may obtain a copy of the standard EU contractual terms by contacting the Principal Investigator.
- You may withdraw your consent at any time.  If you withdraw your consent, this will not affect the lawfulness or our collecting, use and sharing of Your Study Data up to the point in time that you withdraw your consent. Even if you withdraw your consent, we may still use Your Study Data that has been anonymized so that the data no longer identifies you. In addition, we may use and share Your Study Data that has been pseudonymized (by removal of your name and certain other identifiers so that the data does not directly identify you) as permitted by applicable law for purposes of: (a) public health (e.g., ensuring high standards quality and safety of health care), (b) scientific or historical research or statistical analysis as permitted by applicable European Union or European Union Member State laws and (c) archiving in the public interest.

**TIME INVOLVEMENT:** Your participation will take approximately *15 minutes.*

**RISKS AND BENEFITS:** The risks associated with this study are *minimal.* The benefits which may reasonably be expected to result from this study are *none.* We cannot and do not guarantee or promise that you will receive any benefits from this study.

**PAYMENTS:** You will receive an equivalent of *$1* as payment for your participation.

**PARTICIPANT'S RIGHTS:** If you have read this form and have decided to participate in this project, please understand your participation is voluntary and you have the right to withdraw your consent or discontinue participation at any time. However, you may not receive payment for an incomplete survey. The alternative is not to participate. You have the right to refuse to answer particular questions. However, you may not receive payment for an incomplete survey. The results of this research study may be presented at scientific or professional meetings or published in scientific journals. You will be entirely anonymous to the study investigator. We do not have access to your private information in any form whatsoever.

**CONTACT INFORMATION:**

*Questions:* If you have any questions, concerns or complaints about this research, its procedures, risks and benefits, contact the Protocol Director, Dr Maya Adam from Stanford University (email address: madam@stanford.edu).

*Independent Contact:* If you are not satisfied with how this study is being conducted, or if you have any concerns, complaints, or general questions about the research or your rights as a participant, please contact the Stanford Institutional Review Board (IRB) to speak to someone independent of the research team at 650-723-2480 or email at IRB2-Manager@lists.stanford.edu, or toll free at 1-866-680-2906. You can also write to the Stanford IRB, Stanford University, 1705 El Camino Real, Palo Alto, CA 94306.

The extra copy of this consent form is for you to keep. Please print a copy of this page for your records.

**Figure S1**. Schedule of enrolment, interventions, and assessments for the study

|  | **STUDY PERIOD** | | |
| --- | --- | --- | --- |
|  | **Enrolment** | **Allocation** | **Post-allocation** |
| **TIMEPOINT** | $-t_{0}$  *Minute 1* | $t_{0}$  *Minute 0* | $t_{1}$  *Minute 1-10* |
| **ENROLMENT:** |  |  |  |
| **Eligibility screen** | × |  |  |
| **Informed consent** | × |  |  |
| **Allocation** |  | × |  |
| **INTERVENTIONS:** |  |  | × |
| **Arm a** |  |  | × |
| **Arm b** |  |  | × |
| **Arm c** |  |  | × |
| **Control** |  |  | × |
| **ASSESSMENTS:** |  |  | × |
| **Questionnaire survey** |  |  | × |
